# Evaluating early intervention in smoldering myeloma clinical trials: a systematic review

**DOI:** 10.1093/oncolo/oyae219

**Published:** 2024-09-05

**Authors:** Apoorva Kakkilaya, Aaron Trando, Edward R Scheffer Cliff, Hira Mian, Samer Al Hadidi, Muhammad Aziz, Aaron M Goodman, Ah-Reum Jeong, Wade L Smith, Amar H Kelkar, David A Russler-Germain, Nikita Mehra, Rajshekhar Chakraborty, Morie A Gertz, Ghulam Rehman Mohyuddin

**Affiliations:** John Sealy School of Medicine, University of Texas Medical Branch, Galveston, TX, United States; School of Medicine, University of California San Diego, La Jolla, CA, United States; Program on Regulation, Therapeutics and Law, Division of Pharmacoepidemiology and Pharmacoeconomics, Brigham and Women’s Hospital, Harvard Medical School, Boston, MA, United States; Division of Hematology, McMaster University, Hamilton, Canada; Myeloma Center, Winthrop P. Rockefeller Cancer Institute, University of Arkansas for Medical Sciences, Little Rock, AR, United States; Division of Gastroenterology and Hepatology, University of Toledo, Toledo, OH, United States; Division of Blood and Marrow Transplantation, University of California San Diego, La Jolla, CA, United States; Division of Blood and Marrow Transplantation, University of California San Diego, La Jolla, CA, United States; Mulford Health Science Library, University of Toledo, Toledo, OH, United States; Department of Medical Oncology, Dana-Farber Cancer Institute, Harvard Medical School, Boston, MA, United States; Division of Oncology, Washington University School of Medicine, St. Louis, MO, United States; Department of Medical Oncology, Cancer Institute (WIA), Chennai, India; Herbert Irving Comprehensive Cancer Center, Columbia University Irving Medical Center, New York, NY, United States; Division of Hematology, Mayo Clinic, Rochester, MN, United States; Division of Hematology, Huntsman Cancer Institute, University of Utah, Salt Lake City, UT, United States

**Keywords:** smoldering myeloma, myeloma, precursor condition, lenalidomide, systematic review, publication bias

## Abstract

**Background:**

Smoldering multiple myeloma (SMM), an asymptomatic precursor of multiple myeloma (MM), carries a variable risk of progression to MM. There is little consensus on the efficacy or optimal timing of treatment in SMM. We systematically reviewed the landscape of all clinical trials in SMM. We compared the efficacy of treatment regimens studied in SMM to results from these regimens when used in newly diagnosed multiple myeloma (NDMM), to determine whether the data suggest deeper responses in SMM versus NDMM.

**Methods:**

All prospective interventional clinical trials for SMM, including published studies, meeting abstracts, and unpublished trials listed on ClinicalTrials.gov up to April 1, 2023, were identified. Trial-related variables were captured, including treatment strategy and efficacy results. Relevant clinical endpoints were defined as overall survival (OS) and quality of life.

**Results:**

Among 45 SMM trials identified, 38 (84.4%) assessed active myeloma drugs, while 7 (15.6%) studied bone-modifying agents alone. Of 18 randomized trials in SMM, only one (5.6%) had a primary endpoint of OS; the most common primary endpoint was progression-free survival (*n* = 7, 38.9%). Among 32 SMM trials with available results, 9 (28.1%) met their prespecified primary endpoint, of which 5 were single-arm studies. Six treatment regimens were tested in both SMM and NDMM; 5 regimens yielded a lower rate of very good partial response rate or better (≥VGPR) in SMM compared to the corresponding NDMM trial (32% vs 63%, 43% vs 53%, 40% vs 63%, 86% vs 89%, 92% vs 95%, and 94% vs 87%, respectively).

**Conclusion:**

In this systematic review of all prospective interventional clinical trials in SMM, we found significant variability in trial design, including randomization status, primary endpoints, and types of intervention used. Despite the statistical limitations, comparison of treatment regimens revealed no compelling evidence that the treatment is more effective when introduced early in SMM compared to NDMM.

## Teaser

There is little consensus on the efficacy or optimal timing of treatment in smoldering multiple myeloma (SMM). This article reviews the landscape of all clinical trials in this disease, comparing the efficacy of treatment regimens in SMM to results from these regimens when used in newly diagnosed MM.

**Implications for Practice**

Our findings suggest that at this time, given changes to imaging and diagnostic classification, there is very limited randomized evidence that support the treatment of SMM, outside of well-designed clinical trials. Further randomized studies, ideally with an observation control arm, that are powered for appropriate primary endpoints (eg, overall survival and quality of life) are warranted to better understand potential benefits or risks of early intervention in SMM.

## Introduction

Smoldering multiple myeloma (SMM) is a precursor condition to multiple myeloma (MM), occurring in approximately one in 200 individuals over the age of 40.^1^ SMM carries a highly variable risk of progressing to MM. Historical studies suggested that approximately 50% of patients with SMM would develop MM within 5 years of diagnosis. However, this is likely an overestimate due to advances in imaging and the 2014 International Myeloma Working Group (IMWG) diagnostic reclassification, which increased the number of people diagnosed with MM.^2-4^ Although MM is often considered incurable, dramatic advances in treatment raise the question of whether cure may be achievable with novel agents.^5,6^ In some solid cancers, detection at an earlier stage leading to intervention—especially surgery—can lead to cure, such as in colorectal cancer.^7,8^ Similarly, it has been hypothesized that early treatment of SMM before progression to MM may either avert morbidity associated with MM and/or increase the possibility of cure.^9^

There are various hypotheses that biological characteristics of SMM might make it more susceptible to treatment than MM; it is thus often perceived that a given therapy might be more effective in SMM than MM.^10-12^ Based on the concept of early interception plus the recognition that MM always originates from a precursor state, there is strong interest in early intervention for SMM. For example, the ASCENT trial of aggressive 4-drug therapy for SMM was based on the hypothesis that intense therapy applied at this precursor phase could potentially eradicate the malignant clone and lead to long term remissions or even cure.^10^ Furthermore, theoretically preventing or curing MM before it leads to morbidity, such as fractures and renal failure, would be ideal.

Two prospective trials suggested that progression to MM may be delayed by treating SMM. In the randomized QUIREDEX study of 119 patients with high-risk SMM (HR-SMM), the median time to progression (TTP) was significantly longer in patients treated with lenalidomide and dexamethasone compared to patients who were observed.^13^ In a second, more recent trial of 182 patients, of whom 56 had HR-SMM, the use of lenalidomide reduced the risk of progression of SMM to MM.^14^ However, neither trial was adequately powered to assess overall survival (OS), and their relevance to today’s patients is limited, given that the patients previously diagnosed with highest-risk SMM would now be classified as having MM, and many patients previously thought asymptomatic may now be classified as having MM due to more sensitive imaging.^15,16^ Furthermore, as these trials did not clearly outline the nature of progression events, “progression” could range from an asymptomatic lab change, such as a drop in hemoglobin, to more clinically meaningful issues such as fractures or permanent renal failure. Since these events vary significantly in severity, it remains unclear whether the therapy is preventing or delaying asymptomatic, reversible lab changes or symptomatic, irreversible organ damage. Therefore, whether or not to treat patients with high-risk SMM remains controversial.^17^

It ultimately remains unknown whether treatments that are known to be effective against MM are indeed more effective when used in SMM compared to reserving their use for patients whose disease progresses to MM. Furthermore, there is great variability in the methodology of clinical trials that seek to evaluate early interventions in SMM, making it difficult to incorporate the evidence from these largely single-arm trials into clinical practice.^18,19^ In order to capture both completed and in-progress clinical trials in SMM, we systematically reviewed all prospective clinical trials in SMM. To interrogate the hypothesis that treatment may be more effective when used in SMM, we compared the results of treatment regimens used to treat SMM to that regimen’s corresponding outcomes in newly diagnosed MM (NDMM), wherever applicable and possible.

## Methods

### Search strategy and selection criteria

A comprehensive search strategy to identify prospective trials was constructed in Embase (Embase.com, Elsevier) by a health librarian and a clinician using truncated keywords, phrases, proximity searching and subject headings for SMM, and the CADTH Clinical Trials search filter.^20^ This strategy was translated to MEDLINE, Cochrane Central Register of Controlled Trials, and the Web of Science Core Collection with initial searches performed on November 10, 2022, and a follow-up search of ClinicalTrials.gov on April 1, 2023 (see Supplementary Appendix I for detailed search strategies). There was no start date for our search strategy, as we aimed to include all published and unpublished work on SMM. Thus, the start of our first SMM trial was October 1, 1983. For all SMM studies, a search was done on PubMed and Google Scholar for corresponding prospective trials in NDMM using the same regimen on April 30, 2023. No publication date or language limits were used. All results were exported to EndNote version 20 (Clarivate, Philadelphia, USA); duplicates were removed with EndNote’s duplicate detection algorithms and manual inspection.

Two independent reviewers (A.K. and A.T.) screened all studies with discrepancies resolved by a third reviewer (G.R.M.). This systematic review was performed according to the Preferred Reporting Items for Systematic Reviews and Meta-Analyses (PRISMA) recommendations^21^ and a PROSPERO protocol (CRD42022373951) outlining our search and analysis plan a priori was submitted before search initiation. This study was not submitted for Institutional Review Board approval as it was not human participant research.

Our search strategy included all prospective therapeutic clinical trials from 1980 to April 1, 2023. Other study types were excluded (eg, observational cohort and registry). Only studies with patients meeting the diagnostic criteria for SMM were included, while studies of all other plasma cell dyscrasias such as MM were excluded. Studies also had to evaluate potentially active myeloma drugs (including drugs considered to potentially be active but not proven, such as celecoxib and metformin) or bone-modifying agents; studies of other treatment types were excluded. Results posted on non-peer reviewed sources such as trial registry sites and abstracts from conference proceedings captured via our search strategy were included. Trials that were in progress that did not yet have results but had a clear description of methodology and interventions were included.

### Statistical analysis

Two authors (A.K. and A.T.) extracted the following data using Microsoft Excel (Microsoft, Washington, USA): trial design (randomized or nonrandomized), number of patients, median age, treatment regimen, duration of treatment, length of follow-up, study enrollment period, primary endpoint type and outcomes, overall and treatment-related deaths, funding source, and study location. Clinical endpoints were defined according to FDA guidelines as overall survival and symptom endpoints (patient-reported outcomes), while surrogate endpoints included overall response rate (ORR), progression-free survival (PFS), TTP, measurable residual disease (MRD), and safety.^22^

The primary objective of our study was to systematically review clinical trials in SMM, ascertaining whether these studies were randomized, whether they met their prespecified primary endpoint (that the study was statistically powered to assess), and the type of intervention studied, which we categorized as intensive multiagent therapy, preventative low-intensity therapy, or bone-strengthening agents. A key secondary objective was to compare the results of individual regimens used to treat SMM to the corresponding outcomes of that regimen when used in NDMM. The prospective trial in NDMM that had the greatest similarity in dosing and administration to the comparable trial in SMM was used for comparison. We descriptively compared rates of very good partial response or greater (≥VGPR), and also collected ORR and complete response rates for each regimen. We chose to compare response rates and MRD rates rather than PFS, as progression is defined differently in MM (defined by formal progression criteria based on laboratory values and/or end-organ damage), in contrast to SMM, where it is often defined as progression to MM.^4^ Furthermore, time-to-event endpoints such as PFS in SMM can incorporate lead-time bias due to earlier diagnosis.

All completed randomized trials were assessed for bias using the Cochrane Handbook for Systematic Review of Interventions, version 6.2 and Cochrane risk-of-bias tool,^23,24^ and all completed nonrandomized trials were appraised across all domains of bias using the Cochrane Collaboration Risk of Bias in Nonrandomized Studies of Intervention (ROBINS-I) tool.^25^

## Results

**Figure 1.**
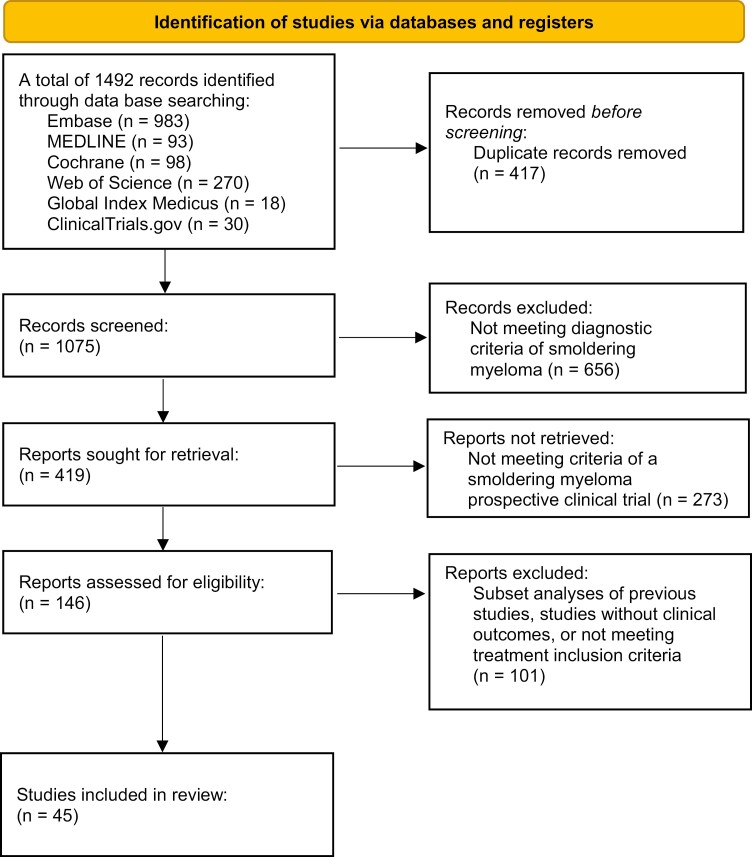
Flow diagram depicting search strategy and study inclusion.

From an initial search finding 1492 results, 45 studies^10,11,13,14,26-66^ were included after excluding duplicates and studies not meeting SMM diagnostic or treatment inclusion criteria (Figure 1). Most trials studied active myeloma drugs (*n* = 38, 84.4%)^10,11,13,14,26-29,32,33,35-39,41-52,54,56-65^ while the remainder studied bone-modifying agents (*n* = 7, 15.6%; Table 1).^30,31,34,40,53,55,66^

**Table 1.** Characteristics of included studies.

Characteristic (*n* = 45)	No. of trials (%)
Study status	
Completed	23 (51.1)
Active, not recruiting, preliminary results available	7 (15.6)
Recruiting, preliminary results available	2 (4.4)
Active, not recruiting, no results available	2 (4.4)
Recruiting, no results available	11 (24.4)
Study design	
Randomized	18 (40.0)
Blinded (amongst randomized)	4 (22.2)
Treatment control arm (amongst randomized)	9 (50.0)
Observation control arm (amongst randomized)	9 (50.0)
Nonrandomized	27 (60.0)
Single-arm	23 (51.1)
Observation control arm	1 (2.2)
Two-group study	2 (4.4)
Multi-group study	1 (2.2)
Median sample size (IQR)	54 (27.5-104.5)
Randomized studies	102 (51-177)
Nonrandomized studies	38 (20-61)
Year study started patient enrollment	
Pre-2000	5 (11.1)
2001-2013	16 (35.6)
Post-2014	24 (53.3)
Publication format	
Manuscript	20 (44.4)
Abstract	13 (28.9)
ClinicalTrials.gov only	12 (26.7)
Treatment approach	
Single agent	18 (40.0)
Multiagent	20 (44.4)
Bone-modifying agent	7 (15.6)
Primary endpoint	
Response rate	16 (35.6)
Randomized	3 (16.7)
Nonrandomized	13 (48.1)
PFS	10 (22.2)
Randomized	7 (38.9)
Nonrandomized	3 (11.1)
MRD	3 (6.7)
Randomized	0 (0.0)
Nonrandomized	3 (11.1)
OS	1 (2.2)
Randomized	1 (5.6)
Nonrandomized	0 (0.0)
TTP	3 (6.7)
Randomized	3 (16.7)
Nonrandomized	0 (0.0)
Translational	2 (4.4)
Randomized	0 (0.0)
Nonrandomized	2 (7.4)
M-protein level	3 (6.7)
Randomized	2 (11.1)
Nonrandomized	1 (3.7)
Safety	3 (6.7)
Randomized	0 (0.0)
Nonrandomized	3 (11.1)
Multiple	2 (4.4)
Randomized	2 (11.1)
Nonrandomized	0 (0.0)
Other	2 (4.4)
Randomized	0 (0.0)
Nonrandomized	2 (7.4)
Funding source	
Industry	23 (51.1)
Nonindustry	19 (42.2)
Unknown	3 (6.7)
Location	
USA	29 (64.4)
International including USA	5 (11.1)
Non-USA only	11 (24.4)

Abbreviations: IQR, interquartile range; M-protein, monoclonal protein; MRD, measurable residual disease; OS, overall survival; PFS, progression-free survival; TTP, time to progression.

Eighteen studies (40.0%)^13,14,27-33,50,52,54-56,58,59,64,65^ were randomized trials while 27 studies (60.0%)^10,11,26,34-49,51,53,57,60-63,66^ were nonrandomized trials. Of 18 randomized trials, the most common primary endpoint was PFS (*n* = 7, 38.9%),^14,27,30,31,50,52,56^ followed by ORR (*n* = 3, 16.7%),^59,64,65^ TTP (*n* = 3, 16.7%),^13,29,55^ M-protein level (*n* = 2, 11.1%),^32,58^ and OS (*n* = 1, 5.6%)^54^; 2 studies (11.1%)^28,33^ had multiple primary endpoints. Fourteen (78%) randomized trials^13,14,28-31,33,50,52,54,56,59,64,65^ were open label and 4 (22%)^27,32,55,58^ were blinded. Only one study (5.6%)^54^ had OS as its primary endpoint. Amongst 27 nonrandomized trials, the most common primary endpoint was ORR (*n* = 13, 48.1%),^10,11,35-37,39,40,46-48,60,62,66^ followed by safety (*n* = 3, 11.1%),^42,51,63^ PFS (*n* = 3, 11.1%),^45,49,61^ MRD (*n* = 3, 11.1%),^23,43,57^ a translational outcome (*n* = 2, 7.4%),^38,41^ other outcomes (*n* = 2, 7.4%),^34,53^ and M-protein level (*n* = 1, 3.7%).^44^

Thirty-two studies^10,11,13,14,26-49,53,57,65,66^ had reported results: 23 (71.9%)^13,14,27-45,65,66^ were completed trials with final results available and 9 (28.1%)^10,11,26,46-49,53,57^ were ongoing with only preliminary results. Among these 32 trials, single-agent treatment (*n* = 14, 43.8%),^14,16,27,28,32,35,37-39,41,44,47,48,65^ multiagent approaches (*n* = 12, 37.5%),^13,15,26,29,33,36,42,43,45,46,49,57^ and bone-modifying agents (*n* = 6, 18.8%)^30,31,34,40,53,55,66^ were tested. Most were nonrandomized (*n* = 22, 68.8%).^10,11,26,34-49,53,57,66^Table 2 lists the characteristics of the 10 (31.3%) completed randomized trials^13,14,27-33,65^ with final results available, including primary outcome results.

**Table 2.** Characteristics of completed randomized SMM trials.

Author, year	Trial name/NCT number	Total number of patients	Study design	Intervention regimen	Control regimen	Primary endpoint	Primary endpoint met?	Primary endpoint results (intervention vs control)
Mateos et al, 2013^13^	QUIREDEX NCT00480363	119	Parallel assignment	Lenalidomide + dexamethasone	Observation	TTP	YES	After a median follow-up time of 12.5 years (range: 10.4-13.6), the median TTP to MM was 2.1 years vs 9.5 years (HR: 0.28, 95% CI: 0.18-0.44, *P* < .0001)
Brighton et al, 2019^27^	NCT01484275	74	Parallel assignment; double-blinded	Siltuximab	Placebo	PFS	NO	The 1-year PFS rate was 84.5% [95% CI, 68.6-92.8] vs 74.4% (95% CI, 57.3-85.5). The study did not meet the prespecified hypothesis that siltuximab would increase the 1-year PFS rate by at least 14%
Landgren et al, 2020^28^	CENTAURUS NCT02316106	123	Parallel assignment, open label	Daratumumab (intense, intermediate, and short)	NA	Co-primary endpoints (CR and PD/death rate per patient-year)	NO	With longer follow-up (median follow-up, 25.9 months), CR rates were 4.9%, 9.8%, and 0% for intense, intermediate, and short dosing, respectively. PD/death rates were 0.059 (80% CI, 0.025-0.092), 0.107 (80% CI, 0.058-0.155), and 0.150 (80% CI, 0.089-0.211), translating to a median PFS ≥ 24 months in all arms (*P* < .0001 for all arms)
Witzig et al, 2013^29^	NCT00432458	68	Parallel assignment, open label	Thalidomide and zoledronic acid	Zoledronic acid	TTP	YES	Median TTP was 2.4 years (95% CI: 1.4-3.6) vs 1.2 years (95% CI: 0.7-2.5; HR, 2.05; 95% CI: 1.1-3.8; *P*-value: .02)
D’arena et al, 2011^30^	NA	197	Parallel assignment, open label	Pamidronate	Observation	PFS	NO	With a minimum follow-up of 5 years for living patients, there were 56/89 (62.9%) progressions vs 55/88 (62.5%; *P* = NS). Median TTP was 46 and 48 months, respectively (*P* = NS)
Musto et al,^31^ 2008	EUCTR2006-003854-33-IT	163	Parallel assignment, open label	Zoledronic acid	Observation	PFS	NO	After a median follow-up of 64.7 person-months, 44.4% of patients in the intervention group and 45.1% of the control group progressed to “symptomatic” myeloma requiring chemotherapy (*P* = .9307)
Horwitz et al, 2012^32^	NCT00099047	23	Parallel assignment, double blind	Celecoxib	Placebo	M-protein levels	NO	Celecoxib had no significant effect on the median monoclonal protein concentration, which went from a median of 1.44-1.65 g/dL with placebo and 2.42-2.24 g/dL with celecoxib (*P* = .36)
Lonial et al, 2020^14^	NCT01169337	182	Parallel assignment, open label	Lenalidomide	Observation	PFS	YES	PFS was significantly longer with lenalidomide compared with observation (HR, 0.28; 95% CI, 0.12-0.62; *P* = .002). 1-, 2-, and 3-year progression-free survival was 98%, 93%, and 91% vs 89%, 76%, and 66%, respectively
NA	KIROMONO NCT01222286	30	Parallel assignment, open label	IPH2101 0.2 mg/kg or IPH2101 2 mg/kg	NA	Response	NO	0% of patients achieved an objective response with either dose of IPH2101
Hjorth et al, 1993^33^	NA	50	Assignment based on disease classification	Initial melphalan-prednisone	Observation until disease progression then melphalan-prednisone	Multiple (response rate, plateau phase/response duration, and overall survival)	NO	Response rate: response was seen in 13 out of 25 (52%) patients vs 12 out of 22 (55%). There was no difference in survival between the 2 treatment groups. Response duration: the duration of relapse-free survival after the cessation of treatment was similar in both groups (*P* = .17; median duration 21 months vs 31 months). Survival: there was no difference in survival between the 2 treatment groups

Abbreviations: CR, complete response; HR, hazard ratio; NA, not available; NS, not significant; M-protein, monoclonal protein; PD, progressive disease; PFS, progression-free survival; TTP, time to progression.

Among 32 trials with results available, 9 studies (28.1%)^13,14,26,29,37,38,42,43,48^ met their prespecified primary endpoint while 18 studies (56.3%)^10,27,28,30-35,39-41,44,46,47,53,65,66^ had not; 2 (6.3%)^49,57^ had not yet reported primary results, and 3 (9.4%)^11,36,45^ had unclear primary outcome results. Among 9 trials that met their primary endpoint, 7 (77.8%)^13,14,29,37,38,42,43^ were published in manuscripts and 2 (22.2%)^26,48^ in abstracts. Of the remaining 23 trials, 16 (69.6%)^27,28,30-36,39-41,44,45,65,66^ have been completed without clear evidence of having met their primary endpoint. Of these 16 trials, 11 (68.8%)^27,28,30,31,33,35,36,39,41,44,66^ published in manuscripts, 4 (25.0%)^32,34,40,45^ in abstract form, and one (6.3%)^65^ on ClinicalTrials.gov only.

Of 13 ongoing studies^50-52,54-56,58-64^ without results available, 8 (61.5%)^50,52,54-56,58,59,64^ are randomized. The majority of these 8 randomized studies list surrogate markers as the primary endpoint, including PFS (*n* = 3, 37.5%),^50,52,56^ ORR (*n* = 2, 25.0%),^59,64^ TTP (*n* = 1, 12.5%),^55^ and M-protein level (*n* = 1, 12.5%).^58^

Among 8 SMM ongoing randomized clinical trials without available results, 5^52,54,56,59,64^ use active control arms (lenalidomide plus dexamethasone in NCT04270409, NCT03673826, NCT03937635, NCT05469893 and iberdomide in NCT04776395) whereas 3 ongoing trials assign observation or placebo to the control arm (NCT03301220, NCT04850846, and NCT03792763).

The median sample size of 10 completed randomized studies was 102 patients (IQR 55-153); of these trials, 4 (40.0%)^28,31,32,65^ had a low overall risk of bias, 3 (30.0%)^14,27,29^ had some concerns, and 3 (30.0%)^13,30,33^ studies had a high risk of bias, according to the Cochrane risk-of-bias tool (Supplementary Appendix II). Of 13 completed nonrandomized trials,^34–45,66^ median sample size was 31 patients (IQR 22-50). The majority (*n* = 10, 76.9%)^36–41,43–45,66^ had a moderate risk of bias, 2 studies^35,42^ were at serious risk of bias, and one study^34^ had insufficient information to allow appraisal (Supplementary Appendix III).

**Figure 2.**
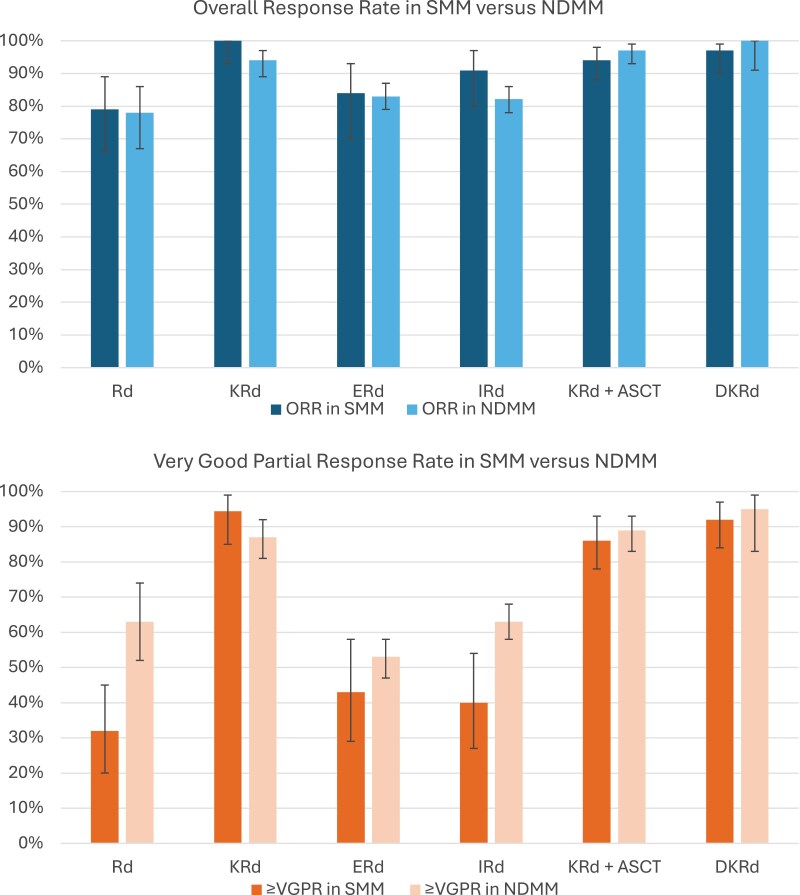
Comparison of overall response rate and very good partial response (or greater) rate in corresponding SMM versus NDMM trials. Abbreviations: ASCT, autologous stem cell transplant; DKRd, daratumumab-carfilzomib-lenalidomide-dexamethasone; ERd, elotuzumab-lenalidomide-dexamethasone; IRd, ixazomib-lenalidomide-dexamethasone; KRd, carfilzomib-lenalidomide-dexamethasone; NDMM, newly diagnosed multiple myeloma; ORR, overall response rate; Rd, lenalidomide-dexamethasone; SMM, smoldering multiple myeloma; VGPR, very good partial response.

Among 6 treatment regimens with ≥VGPR data available in both SMM and NDMM settings (Table 3; Supplementary Appendix IV), 5 regimens yielded a lower ≥VGPR in SMM compared to NDMM: lenalidomide plus dexamethasone (32% in SMM vs 63% in NDMM); elotuzumab, lenalidomide and dexamethasone (43% vs 53%); ixazomib, lenalidomide and dexamethasone (40% vs 63%); carfilzomib, lenalidomide, dexamethasone, autologous stem cell transplant (ASCT; 86% vs 89%); and daratumumab, carfilzomib, lenalidomide and dexamethasone without ASCT (92% vs 95%; Figure 2); whereas one regimen showed the reverse: carfilzomib, lenalidomide and dexamethasone without ASCT (94% in SMM vs 87% in NDMM). MRD results were sparsely and heterogeneously assessed^73^; however, of the 3 regimens with MRD-negativity rates available in both SMM and NDMM settings, one regimen (daratumumab, carfilzomib, lenalidomide and dexamethasone without ASCT) had higher MRD-negativity rates in SMM, one (carfilzomib, lenalidomide, dexamethasone, and ASCT) had higher MRD-negativity rates in NDMM, and the other (carfilzomib, lenalidomide, and dexamethasone) had similar rates in both settings.^10,26,43,68,71^

**Table 3.** Comparison of regimen efficacy in smoldering myeloma versus newly diagnosed myeloma.

Regimen	NCT number of smoldering trial and NDMM trial	Dosing in smoldering myeloma trial	Dosing in multiple myeloma trial	ORR in SMM	ORR in NDMM	≥VGPR in SMM	≥VGPR in NDMM	≥CRR in SMM	≥CRR in NDMM	MRD data in SMM	MRD data in NDMM
Lenalidomide-dexamethasone	NCT00480363 NCT00064038	Len = 25 mg (days 1-21), dex 20 mg (days 1-4, 12-15, and 12-15) for nine 28-day cycles, then maintenance len = 10 mg (days 1-21) for 2 years^^13^^	Len = 25 mg (days 1-28), dex 40 mg (days 1-4, 9-12, and 17-20) of 28-day cycle for 3 cycles, followed by maintenance of dex 40 mg (days 1-4 and 15-18) and len 25 mg (days 1-21) of 28-day cycle continuously until progression/intolerance^67^	79% (95% CI, 66%-89%) at end of induction with 9 cycles	78% (95% CI, 67%-86%) after one year of therapy	32% (95% CI, 20%-45%)	63% (95% CI, 52%-74%)	21% (95% CI, 11%-34%)	26% (95% CI, 17%-37%)	NR	NR
Lenalidomide	NCT01169337	Len = 25 mg (days 1-21) of 28-day cycle^^14^^	No corresponding trial exists for lenalidomide monotherapy in NDMM	50% (95% CI, 39%-61%) at any point while on therapy during 2 years	NA	4.5% (95% CI, 1%-11%)	NA	0%	NA	NR	NA
Carfilzomib-lenalidomide-dexamethasone (KRd)	NCT01572480 NCT02203643	K = 20/36 mg/m^2^ twice weekly, len = 25 mg days 1-21, dex (20 mg C1-4; C5-8 twice a week) for 8 cycles → Len (10 mg) × 24 cycles^43^	K = 36 mg/m^2^ (first 2 doses at 20mg/m^2^) on days 1, 2, 8, 9, 15, and 16, len = 25 mg days 1-21, dex (20 mg twice a week) for 12 cycles, separate randomization for maintenance^68^	100% (95% CI, 93%-100%) after 8 cycles of therapy	94% (95% CI, 89%-97%) after 1 year of therapy, prior to second randomization	94.4% (95% CI, 85%-99%)	87% (95% CI, 81%-92%)	75.9% (95% CI, 62%-87%)	57% (95% CI, 49%-65%)	70.4% MRD negative CRRs at 10^−5^	69% at 10^−5^ after completion of 12 cycles of KRd
Elotuzumab-lenalidomide-dexamethasone (ERd)	NCT02279394 NCT01335399	Elotuzumab standard dosing^a^, Dex = 40 mg days 1, 8, 15, and 22, cycles 1-2, then 40 mg oral days 1, 8, and 15, cycles 3-8. Len monotherapy continued for 2 years of therapy^^45^^	Elotuzumab standard dosing^a^, Len = 25 mg days 1-21, Dex = 40 mg days 1, 8, 15, and 22 of each cycle until progression/toxicity^69^	84% (95% CI, 70%-93%) at any point within 2 years of therapy	83% (95% CI, 79%-87%) at any point during therapy	43% (95% CI, 29%-58%)	53% (95% CI, 47%-58%)	6% (95% CI, 1%-17%)	18% (95% CI, 14%-22%)	NR	NR
Ixazomib-lenalidomide-dexamethasone (IRd)	NCT02916771 NCT01850524	Ixazomib = 4 mg days 1, 8, and 15, len = 25 mg days 1-21, dex = 40 mg days 1, 8, 15, and 22 of 28-day cycle; dex discontinued after cycle 8, total of 24 cycles^49^	Ixazomib = 4 mg days 1,8,15, len = 25 mg days 1-21, dex = 40 mg days 1, 8, 15, and 22 of 28-day cycle. Dex discontinued and ixazomib/len reduced to 3 mg/10 mg respectively, continued until progression/intolerance^70^	90.9% (95% CI, 80%-97%) at any point during the study treatment period	82.1% (95% CI, 78%-86%) at any point during study treatment period	40% (95% CI, 27%-54%)	63% (95% CI, 58%-68%)	21.8% (95% CI, 12%-35%)	25.6% (95% CI, 21%-31%)	NR	101 patients (28.8% of patients in IRd arm) had MRD evaluated: 52.5% MRD negative at 10^−5^
Carfilzomib-lenalidomide-dexamethasone + ASCT (GEM-CESAR trial, KRd-ASCT)	NCT02415413 NCT02203643	K = 20/36 mg/m^2^ twice weekly, len = 25 mg (days 1-21), dex = 40 mg weekly for six 28-day cycles → Mel-200/ASCT → KRD x 2 cycles →Rd × 2 years, (len = 10 mg/day, dex = 20 mg/week)^26^	Carfilzomib = 36 mg/m^2^ (first 2 doses = 20 mg/m^2^) days 1, 2, 8, 9, 15, and 16, len = 25 mg (days 1-21), dex = 20 mg twice a week for four 28-day cycles → Mel-200/ASCT→ KRD × 2 cycles → randomization for maintenance^^68^^	94% (95% CI, 88%-98%) after end of consolidation therapy	97% (95% CI, 93%-99%) after end of consolidation therapy	86% (95% CI, 78%-93%)	89% (95% CI, 83%-93%)	70% (95% CI, 61%-80%)	54% (95% CI, 46%-62%)	63% MRD neg rates at 10^−5^ postASCT 23% sustained MRD at 10^−6^ 4 years after ASCT	80% MRD neg rates at 10^−5^ post ASCT
Daratumumab-carfilzomib-lenalidomide-dexamethasone without transplant (ASCENT trial, DKRd)	NCT03289299 NCT03290950	K = 20/56 mg/m^2^ once weekly, len = 25 mg days 1-21, dara = 16 mg/kg weekly, dex = 40 mg weekly for 28-day cycles. Dara standard dosing^b^, len dose reduced and dex discontinued after 12 cycles; total 2 years therapy^10^	Carfilzomib = 56 mg/m^2^ days 1, 8, and 15, len = 25 mg days 1-21, dara = 16 mg/kg weekly, dex = 40 mg weekly for 8 cycles.^71^	97% (95% CI, 90%-99%) at any point during study treatment period	100% (95% CI, 91%-100%) after 8 cycles of therapy	92% (95% CI, 84%-97%)	95% (95% CI, 83%-99%)	63% (95% CI, 52%-73%)	NR	84% at 10^−5^ At median time to MRD negativity of 6.6 months	71% at 10^−5^ after 8 cycles of therapy
Daratumumab-lenalidomide-bortezomib-dexamethasone without transplant (DVRd)	NCT04775550	Dara standard dosing^b^, bortezomib days 1, 8, and 15 for cycles 1-6 and then biweekly until completion of cycle 24. Len is administered days 1-21 and dex is administered weekly until cycle 24^72^	No corresponding trial exists in NDMM	90% (95% CI, 68%-99%) at any point during study treatment period	NA	50% (95% CI, 27%-73%)	NA	25% (95% CI, 8%-49%)	NA	50% at 10^−5^ 25% at 10^−6^ after≥6 months follow-up, in those for whom MRD assessed	NA

Abbreviations: ASCT, autologous stem cell transplant; C, cycle; CRR, complete response rate; Dara (D), daratumumab; Dex (d), dexamethasone; E, elotuzumab; I, ixazomib; K, carfilzomib; Len (R), lenalidomide; Mel, melphalan; MRD; measurable residual disease; NA, not available; NCT, national clinical trial; NDMM, newly diagnosed multiple myeloma; NR, not reported; ORR, overall response rate; SMM, smoldering multiple myeloma; VGPR, very good partial response.

^a^Elotozumab standard dosing is as follows; cycles 1 and 2: 10 mg/kg IV once a week on days 1, 8, 15, and 22 of a 28-day cycle, cycle 3 and beyond: 10 mg/kg IV once every 2 weeks on days 1 and 15 of a 28-day cycle until disease progression or unacceptable toxicity.

^b^Daratumumab standard dosing is weekly for first 8 weeks, every 2 weeks for next 16 weeks, and then monthly.

Of 45 total studies, 8 studies^11,26,32,34,40,45,49,65^ that had primary endpoint results available either on ClinicalTrials.gov or in abstract format by December 2021 (a cutoff date that was chosen to allow time for data analysis and publication) remained unpublished by April 2023. These 8 studies were then further described (Supplementary Appendix V); 7 out of 8 (87.5%)^11,32,34,40,45,49,65^ either did not meet their primary endpoint or did not clearly specify whether they met their primary endpoint.

The mode of progression to MM (biochemical vs clinical) was inconsistently described across studies, precluding quantitative analysis. Of 32 studies with results available, only 7 studies (21.9%)^14,28,30,31,34,43,66^ detailed the nature of progression (Supplementary Appendix VI).

## Discussion

In this systematic review of clinical trials in SMM, we find that most SMM trials are single-arm, nonrandomized studies not adequately powered to assess clinically meaningful endpoints. Surrogate endpoints were the primary endpoint for almost all (97.7%) trials. Furthermore, nearly three-quarters of SMM studies with reported results failed to meet their primary endpoint or did not clearly report whether the primary endpoint was met. Seven studies that did not meet their endpoint have not been published at the time of our analysis, suggesting possible publication bias.

Importantly, when a regimen has been assessed in both SMM and NDMM contexts, response rates were similar, suggesting there is an absence of convincing data to support the hypothesis that SMM is “more responsive” to therapy than MM, even when considering the limitations of cross-trial comparison. We also find that most of these studies have at least a moderate risk of bias, further reinforcing the need for high-quality randomized studies in SMM.

Early intervention for SMM is based on the premise that lower-disease burden may be more responsive to treatment, and thus more curable, than symptomatic disease (ie, frank MM).^10,26^ So far, only one randomized trial in SMM has been powered to detect an improvement in overall survival; this trial is ongoing, with no results available yet (NCT03937635). For an asymptomatic condition such as SMM that has variable risk of progression to symptomatic disease (ie, some patients never progress to MM), it is unclear whether trial endpoints based on response or depth of response (eg, MRD negativity) are meaningful for patients. Notably, even in NDMM, the endpoints of ORR and PFS correlate poorly with OS.^74,75^ Furthermore, treatment is often accompanied by impactful side effects^9^ and is expensive, which can lead to financial toxicity for patients^76^; given that in the United States, lenalidomide can cost >$17 000 a month per patient and quadruplet regimens can range from $300 000 to $500 000/year, MM is one of the most expensive cancers to treat in terms of drug costs.^18,77,78^

We did not find convincing evidence that SMM is inherently more responsive to therapy than MM. Differences in study populations, dosing schema, and duration of therapy precluded formal statistical comparison. However, our findings of similar or lower response rates to therapies in SMM versus NDMM challenge existing dogma. One potential explanation could be that SMM clones, which can be slower growing, may be harder to eradicate than MM clones. This is consistent with emerging evidence positing that patients with monoclonal gammopathy of undetermined significance-like phenotype in SMM exhibit low disease progression rates with no difference in TTP between treatment versus observation.^79^ This is also congruent with real world evidence from the Australia and New Zealand Myeloma and Related Diseases Registry of 1818 patients with MM, which showed no difference in response rates between early- and advanced-stage MM.^80^

Universal treatment of high-risk SMM overtreats many who would do well with just observation alone. For example, even among patients with high-risk SMM in the observation arm of the E3A06 trial, at least 50% were free from progression at 3 years.^14^ Seminal work by Kyle et al defining the natural history of SMM over 15 years also showed that even at 15 years, 27% of patients remained without disease progression.^81^ Furthermore, there is a current lack of reporting of the breakdown of CRAB progression in the majority of SMM trials, making it difficult to ascertain what effects early treatment may avert. In a recent retrospective cohort study, most progression events were asymptomatic laboratory changes, rather than morbid events such as fracture or renal failure.^82^ To justify the treatment of these patients, a net benefit must therefore be demonstrated in a randomized trial powered to assess a clinically-meaningful endpoint.

Current risk stratification models poorly predict patients’ risk of progression and have poor concordance with each other.^82,83^ Evolving models that incorporate changes to lab markers over time and genomics may help improve prediction of which patients are more likely to progress and need therapy.^84^ While it is paramount to longitudinally collect biospecimens as part of SMM trials for future translational research, the benefits to an individual study participant for enrolling on a therapeutic trial may not outweigh the risks of the interventions being offered.

The lack of SMM studies with active surveillance as the control arm not only makes it difficult to know whether patients ultimately benefit from earlier treatment, but also to capture potential harms of early intervention, which is especially important for asymptomatic patients.^9^ This was highlighted by patient deaths in recent trials of intensive therapy for SMM: GEM-CESAR^26^ (7 deaths, 8% of patients; and 51% of patients with nonhematological grade ≥3 toxicities) and ASCENT^13^ (3 deaths, 3% of patients). The lack of a control arm in these studies makes it impossible to know whether these deaths were due to treatment, and whether these patients would have lived longer if they had instead been observed until progression. Similarly, while there is an emergence of targeted therapies suggesting promising activity, such as the recent Immuno-PRISM^85^ trial of teclistamab (*n* = 19, ORR = 100%, MRD negativity = 100% at 10^−6^ for the 8 evaluable patients), the lack of a control arm receiving no therapy presents 2 major unanswered counterfactuals. First, we do not know how these patients would have responded if treated at the time of progression to myeloma and second, due to the lack of a control arm receiving active surveillance, we do not know how patients would have done without therapy. Patients in the trial arm (*n* = 12) experienced grade 3 or higher toxicities such as neutropenia (*n* = 4, 33.3%), pancreatitis (*n* = 1, 8.3%), elevated ALT (*n* = 3, 25%). The duration of response is also unknown. In comparison, a prospective observational cohort study of 96 patients with SMM observed no non-myeloma-related deaths after 28 months median follow-up.^86^

As treatment options improve for MM, it becomes increasingly difficult to show an overall survival benefit in newly diagnosed MM, let alone SMM. Nevertheless, we believe that the bar for adopting early intervention in an asymptomatic population should be an improvement in overall survival or quality of life with the adoption of the intervention. As an example, in asymptomatic chronic lymphocytic leukemia, agents have shown a PFS benefit, but no OS benefit, and the field has not adopted early therapy despite these data.^87^ The neutral prior has been that precursor hematological conditions that are not causing morbidity in their current state should not be treated unless earlier treatment improves survival.

We recognize that each hematological malignancy is different. For instance, the discovery of a high-grade lymphoma, even if asymptomatic, represents a very different clinical scenario than the discovery of an asymptomatic low-grade follicular lymphoma. Given the long follow-up required to ascertain overall survival, using an endpoint that captures the nature of progression—such as distinguishing between asymptomatic lab changes and morbid end-organ damage—may be a useful intermediate. This approach is being explored in a prospective noninterventional SMM trial (NCT06212323).^88^

### Limitations

Limitations of this study include that assessed studies may only have had limited follow-up and many were limited by sample size. Furthermore, although we do not make claims that one regimen is more effective than another, or more effective in one context than another, comparison across trials (between NDMM and SMM) should be considered descriptive only and therefore interpreted with caution. We analyzed all studies in SMM, including studies done prior to diagnostic reclassification and the use of advanced imaging, and these studies may not be relevant to modern day SMM.^89^ Prospective studies are needed to evaluate the natural history of contemporary SMM, such as the SPOTLIGHT study (NCT06212323).

## Conclusions

Our study highlights the heterogeneity of clinical trials evaluating interventions in SMM and suggests there is a lack of evidence to suggest that treatment regimens are more effective in SMM than in NDMM. Randomized trials powered to assess clinically meaningful endpoints (especially overall survival), with active surveillance as their control arm, are needed to assess the risk-benefit relationship in contemporary patients with SMM.

## Supplementary Material

oyae219_suppl_Supplementary_Appendixes

## Data Availability

All data included in this manuscript are publicly available from the original publications from which data were extracted.
