# Supplementary material for: Evaluating early intervention in smoldering myeloma clinical trials: a systematic review
Source: Oncologist. 2024 Sep 5;30(2):oyae219. doi: 10.1093/oncolo/oyae219 (PMC11883161; doi:10.1093/oncolo/oyae219)
Supplement: oyae219_suppl_Supplementary_Appendixes [file oyae219_suppl_supplementary_appendixes.pdf]

Supplement Table of Contents

	Page Number
**Appendix I.** Supplementary Information: Full Search Strategies (all searches performed November 10, 2022 unless otherwise indicated).	2-4
**Appendix II.** Quality Assessment of Completed Randomized Controlled Studies.	5-6
**Appendix III.** Quality Assessment of Completed Non-Randomized Clinical Trials.	7-8
**Appendix IV.** Study Characteristics of Regimens in Smoldering Multiple Myeloma (SMM) and Newly-Diagnosed Multiple Myeloma (NDMM).	9
**Appendix V:** Studies With Results Presented in Abstract Form or on ClinicalTrials.gov with No Manuscript Published to Date.	10
**Appendix VI.** Studies Detailing CRAB Progression.	11

Appendix I. Supplementary Information: Full Search Strategies (all searches performed November 10, 2022 unless otherwise indicated).

Embase (Embase.com, Elsevier)

No.	Query	Results
#1	'smoldering myeloma*' OR 'smoldering multiple myeloma*' OR 'smouldering myeloma*' OR 'smouldering multiple myeloma*' OR 'asymptomatic myeloma*' OR 'indolent myeloma*' OR 'smoldering multiple myeloma'/exp	2039
#2	#1 NOT ([animals]/lim NOT [humans]/lim) NOT ('conference review'/it OR 'editorial'/it OR 'letter'/it OR 'note'/it OR 'review'/it OR 'short survey'/it OR 'tombstone'/it OR 'case report'/de OR 'meta analysis'/de OR 'meta analysis topic'/de OR 'systematic review'/de OR 'systematic review topic'/de)	1356
#3	#2 AND ('randomized controlled trial':de OR 'controlled clinical trial':de OR 'pragmatic clinical trial':de OR 'clinical study':de OR 'adaptive clinical trial':de OR 'equivalence trial':de OR 'clinical trial':de OR 'clinical trial, phase i':de OR 'clinical trial, phase ii':de OR 'clinical trial, phase iii':de OR 'clinical trial, phase iv':de OR 'clinical trial protocol':de OR 'multicenter study':de OR 'clinical trial'/exp OR 'clinical trials as topic'/exp OR 'clinical trial protocol'/exp OR 'clinical trial protocols as topic'/exp OR 'clinical trial (topic)'/exp OR 'clinical studies as topic'/de OR 'multicenter study'/de OR 'multicenter studies as topic'/de OR 'multicenter study (topic)'/de OR 'randomization'/de OR 'random allocation'/de OR 'double-blind method'/de OR 'double blind procedure'/de OR 'double-blind studies'/de OR 'single-blind method'/de OR 'single blind procedure'/de OR 'single-blind studies'/de OR 'placebos'/de OR 'placebo'/de OR 'control groups'/de OR 'control group'/de OR 'cross-over studies'/de OR 'crossover procedure'/de OR random*:ti,ab,kw,de OR sham:ti,ab,kw,de OR placebo*:ti,ab,kw,de OR allocated:ti,ab,de OR nonrandom*:ti,ab,kw,de OR 'non random*':ti,ab,kw,de OR 'quasi random*':ti,ab,kw,de OR quasirandom*:ti,ab,kw,de OR ((singl* OR doubl*) NEAR/2 (blind* OR dumm* OR mask*)) OR ((tripl* OR trebl*) NEAR/2 (blind* OR dumm* OR mask*)) OR (control* NEAR/4 (study OR studies OR trial* OR group*)) OR (clinical NEAR/4 (study OR studies OR trial*)) OR (phase NEAR/4 (study OR studies OR trial*)) OR ((crossover OR 'cross over') NEAR/4 (study OR studies OR trial*)) OR ('open label' NEAR/6 (study OR studies OR trial*)) OR ((equivalence OR superiority OR 'non inferiority' OR noninferiority) NEAR/4 (study OR studies OR trial*)) OR 'pragmatic stud*' OR ((pragmatic OR practical) NEAR/4 trial*) OR ((quasiexperimental OR 'quasi experimental') NEAR/4 (study OR studies OR trial*)) OR ((multicent* OR 'multi cent*') NEAR/4 (study OR studies OR trial*))) NOT (('animals'/exp OR 'animal experimentation'/exp OR 'models animal'/exp OR 'animal experiment'/exp OR 'nonhuman'/exp OR 'vertebrate'/exp) NOT ('humans'/exp OR 'human experiment'/exp))	983

MEDLINE (Ovid MEDLINE(R) ALL 1946 to November 10, 2022, National Center for Biotechnology Information, National Library of Medicine)

No.	Query	Results
#1	exp "Smoldering Multiple Myeloma"/ or Smoldering-myeloma*.mp. or smoldering-multiple-myeloma*.mp. or Smouldering-myeloma*.mp. or smouldering-multiple-myeloma*.mp. or asymptomatic-myeloma*.mp. or indolent-myeloma*.mp. [mp=title, book title, abstract, original title, name of substance word, subject heading word, floating sub-heading word, keyword heading word, organism supplementary concept word, protocol supplementary concept word, rare disease supplementary concept word, unique identifier, synonyms, population supplementary concept word, anatomy supplementary concept word]	757
#2	#1 not (exp "animals"/ not exp "humans"/) not ("case reports" or "comment" or "editorial" or "guideline" or "introductory journal article" or "meta analysis" or "news" or "retracted publication" or "review" or "systematic review").pt.	404
No.	Query	Results

#3	#2 and ((Randomized Controlled Trial or Controlled Clinical Trial or Pragmatic Clinical Trial or Clinical Study or Adaptive Clinical Trial or Equivalence Trial or (Clinical Trial or Clinical Trial, Phase I or Clinical Trial, Phase II or Clinical Trial, Phase III or Clinical Trial, Phase IV or Clinical Trial Protocol) or Multicenter Study).pt. or Clinical Studies as Topic/ or exp Clinical Trial/ or exp Clinical Trials as Topic/ or Clinical Trial Protocol/ or Clinical Trial Protocols as Topic/ or Multicenter Study/ or Multicenter Studies as Topic/ or Randomization/ or Double-Blind Method/ or Single-Blind Method/ or Placebos/ or Control Groups/ or Cross-Over Studies/ or (random* or sham or placebo*).ti,ab,hw,kf. or ((singl* or doubl*) adj (blind* or dumm* or mask*)).ti,ab,hw,kf. or ((tripl* or trebl*) adj (blind* or dumm* or mask*)).ti,ab,hw,kf. or (control* adj3 (study or studies or trial* or group*)).ti,ab,hw,kf. or (clinical adj3 (study or studies or trial*)).ti,ab,hw,kf. or (Nonrandom* or non random* or non-random* or quasi-random* or quasirandom*).ti,ab,hw,kf. or (phase adj3 (study or studies or trial*)).ti,ab,hw,kf. or ((crossover or cross-over) adj3 (study or studies or trial*)).ti,ab,hw,kf. or ((multicent* or multi-cent*) adj3 (study or studies or trial*)).ti,ab,hw,kf. or allocated.ti,ab,hw. or ((open label or open-label) adj5 (study or studies or trial*)).ti,ab,hw,kf. or ((equivalence or superiority or non-inferiority or noninferiority) adj3 (study or studies or trial*)).ti,ab,hw,kf. or (pragmatic study or pragmatic studies).ti,ab,hw,kf. or ((pragmatic or practical) adj3 trial*).ti,ab,hw,kf. or ((quasiexperimental or quasi-experimental) adj3 (study or studies or trial*)).ti,ab,hw,kf. or trial.ti,kf.)	93

Cochrane Central Register of Controlled Trials (CochraneLibrary.com platform, Wiley, Issue 10 of 12, October 2022)

ID	Search	Hits
#1	Smoldering-myeloma* OR smoldering-multiple-myeloma* OR Smouldering-myeloma* OR smouldering-multiple-myeloma* OR asymptomatic-myeloma* OR indolent-myeloma* OR [mh "Smoldering Multiple Myeloma"]	103
#2	98 Trials matching "#1 - Smoldering-myeloma* OR smoldering-multiple-myeloma* OR Smouldering-myeloma* OR smouldering-multiple-myeloma* OR asymptomatic-myeloma* OR indolent-myeloma* OR [mh "Smoldering Multiple Myeloma"]"	98

Web of Science Core Collection (Web of Science Platform, Clarivate, Editions: Arts & Humanities Citation Index, Emerging Sources Citation Index [previous 5 years], Conference Proceedings Citation Index, Science Citation Index-EXPANDED, and Social Science Citation Index)

Search	Hits
Smoldering-myeloma* OR smoldering-multiple-myeloma* OR Smouldering-myeloma* OR smouldering-multiple-myeloma* OR asymptomatic-myeloma* OR indolent-myeloma* (Topic)<br>AND<br>(random* or sham or placebo*) OR ((singl* or doubl*) NEAR/2 (blind* or dumm* or mask*)) OR ((tripl* or trebl*) NEAR/2 (blind* or dumm* or mask*)) OR (control* NEAR/4 (study or studies or trial* or group*)) OR (clinical NEAR/4 (study or studies or trial*)) OR (Nonrandom* or non-random* or quasi-random* or quasirandom*) OR (phase NEAR/4 (study or studies or trial*)) OR ((crossover or cross-over) NEAR/4 (study or studies or trial*)) OR ((multicent* or multi-cent*) NEAR/4 (study or studies or trial*)) OR allocated OR ((open-label) NEAR/6 (study or studies or trial*)) OR ((equivalence or superiority or non-inferiority or noninferiority) NEAR/4 (study or studies or trial*)) OR ((pragmatic-study or pragmatic-studies) OR ((pragmatic or practical) NEAR/4 trial*)) OR ((quasiexperimental or quasi-experimental) NEAR/4 (study or studies or trial*)) OR trial (Topic)<br>AND<br>Review Article or Editorial Material or Case Report or Letter or Reference Material or Patent or Book (Exclude – Document Types) and Web of Science Core Collection (Database)	270

Global Index Medicus (www.globalindexmedicus.net, Includes: African Index Medicus (AIM), Index Medicus for the Eastern Mediterranean Region (IMEMR), Index Medicus for the South-East Asia Region (IMSEAR), Latin America and the Caribbean Literature on Health Sciences (LILACS), and Western Pacific Region Index Medicus (WPRO). World Health Organization)

Search	Hits
tw:((tw:("Smoldering myeloma" OR "smoldering multiple myeloma" OR "Smouldering myeloma" OR "smouldering multiple myeloma" OR "asymptomatic myeloma" OR "indolent myeloma")))	18

Appendix II. Quality Assessment of Completed Randomized Controlled Studies.

Trial ID (Author or NCT Trial Number)	Bias due to randomization process	Bias due to deviations from the intended interventions	Bias due to missing outcome data	Bias due to measurement of the outcome	Bias due to selection of the reported result	Overall risk of bias
Mateos et al,[13] 2013	+	−	+	+	+	−
Lonial et al,[14] 2020	×	+	+	+	+	×
Brighton et al,[27] 2019	+	+	×	+	+	×
NCT01222286	+	+	+	+	+	+
Landgren et al,[28] 2020	+	+	+	+	+	+
Witzig et al,[29] 2013	×	+	+	+	+	×
D'arena et al,[30] 2011	+	×	−	+	+	−
Musto et al,[31] 2008	+	+	+	+	+	+

Trial ID (Author or NCT Trial Number)	Bias due to randomization process	Bias due to deviations from the intended interventions	Bias due to missing outcome data	Bias due to measurement of the outcome	Bias due to selection of the reported result	Overall risk of bias
Horwitz et al,[32] 2012	+	+	+	+	+	+
Hjorth et al,[33] 1993	×	−	+	+	×	−

Low-Risk + Some Concerns × High-Risk − Unknown Risk ?

Appendix III. Quality Assessment of Completed Non-Randomized Clinical Trials.

Trial ID (Author or NCT Trial Number)	Bias due to confounding	Bias in selection of participants into study	Bias in classification of interventions	Bias due to deviations from intended interventions	Bias due to missing data	Bias in measurement of outcomes	Bias in selection of reported result	Overall risk of bias
Kazandjian et al,[43] 2021	+	+	+	+	+	+	+	+
Ghobrial et al,[45] 2017	+	+	+	+	+	+	+	+
Jagannath et al,[41] 2018	+	+	+	+	+	+	+	+
Barlogie et al,[36] 2008	+	+	+	+	+	+	+	+
Nooka et al,[42] 2018	+	+	+	+	+	−	+	−
Munshi et al,[40] 2012	+	+	+	+	+	+	+	+
Carlsten et al,[39] 2016	+	+	+	+	+	+	+	+
Lust et al,[38] 2016	+	+	+	+	+	+	+	+
Wichert et al,[44] 2017	+	+	+	+	+	+	+	+

Trial ID (Author or NCT Trial Number)	Bias due to confounding	Bias in selection of participants into study	Bias in classification of interventions	Bias due to deviations from intended interventions	Bias due to missing data	Bias in measurement of outcomes	Bias in selection of reported result	Overall risk of bias
Frigeri et al,[35] 1995	Low-risk	Low-risk	Low-risk	Low-risk	Serious-risk	Moderate-risk	Low-risk	Serious-risk
Detweiler-Short et al,[37] 2010	Low-risk	Low-risk	Low-risk	Low-risk	Low-risk	Moderate-risk	Low-risk	Moderate-risk
Martin et al,[66] 2002	Low-risk	Low-risk	Low-risk	Low-risk	Low-risk	Moderate-risk	Low-risk	Moderate-risk
Musto et al,[34] 2000	No information	No information	No information	No information	No information	No information	No information	No information

Low-risk + Moderate-risk + Serious-risk − Critical-risk − No information ?

Appendix IV. Study Characteristics of Regimens in Smoldering Multiple Myeloma (SMM) and Newly-Diagnosed Multiple Myeloma (NDMM).

Regimen	NCT Number	Enrollment Years	Randomization Status	Sample size	Median Follow-up Time
**SMM**: Lenalidomide-dexamethasone	NCT00480363	2007-2013	Randomized	57 in treatment group, 62 in observation group	40 months
**NDMM**: Lenalidomide (len)-dexamethasone (dex)	NCT00064038	2004-2012	Randomized, original allocation unblinded	97 in len plus dex, 95 in placebo plus dex	47.2 months
**SMM**: Lenalidomide	NCT01169337	2011-2019	Randomized, open label	92 in treatment group, 90 in observation	35 months
**SMM**: Carfilzomib-lenalidomide-dexamethasone (KRd)	NCT01572480	2012-2020	Nonrandomized	54 patients	31.9 months
**NDMM**: Carfilzomib-lenalidomide-dexamethasone; Carfilzomib-lenalidomide-dexamethasone + autologous stem cell transplant (ASCT) + carfilzomib-lenalidomide maintenance (KRd)	NCT02203643	2015-2016	Randomized, open label	157 patients to KRd, 158 to KRD plus ASCT, 159 to Kd-cyclophosphamide plus ASCT	50.9 months
**SMM**: Elotozumab-lenalidomide-dexamethasone (ERd)	NCT02279394	2014-2018	Nonrandomized	50 patients	65.8 months
**NDMM**: Elotuzumab-lenalidomide-dexamethasone (ERd)	NCT01335399	2011-2019	Randomized	374 in ERd; 374 in len-dex	70.6 months
**SMM**: Ixazomib-lenalidomide-dexamethasone (IRd)	NCT02916771	2016-2025	Nonrandomized	61 patients	Not reported
**NDMM**: Ixazomib-lenalidomide-dexamethasone (IRd)	NCT01850524	2013-2019	Randomized	351 to IRd, 354 to placebo-Rd	53.3 months in IRd, 55.8 months in placebo-Rd arm
**SMM**: Carfilzomib-lenalidomide-dexamethasone + ASCT + carfilzomib-lenalidomide maintenance (KRd) (GEM-CESAR trial)	NCT02415413	2015-2018	Nonrandomized	90 patients	Not reported
**SMM**: Daratumumab-carfilzomib-lenalidomide-dexamethasone without transplant (D-KRd, ASCENT trial)	NCT03289299	2018-2023	Nonrandomized	87 patients	25.8 months
**NDMM**: Daratumumab-carfilzomib-lenalidomide-dexamethasone without transplant (D-KRd)	NCT03290950	2017-2024	Nonrandomized	41 patients	11 months
**SMM**: Daratumumab-lenalidomide-bortezomib-dexamethasone without transplant (DVRd)	NCT04775550	2021-2026	Nonrandomized	20 patients	6 months

Appendix V: Studies With Results Presented in Abstract Form or on ClinicalTrials.gov With No Manuscript Published to Date.

ClinicalTrials.gov Identification Number	Intervention	Primary Endpoint Met?	Year(s) of Abstract Presentation
NCT02279394	Elotuzumab, lenalidomide, and dexamethasone	Unclear	2018
NCT03236428	Daratumumab monotherapy	Unclear	2019, 2021
NCT02916771	Ixazomib, lenalidomide and dexamethasone	Unclear	2021
NCT01222286	IPH2101	No	Results posted on ClinicalTrials.gov in 2014, manuscript not published
NCT00099047	Celecoxib	No	2012
NCT01302886	BHQ880	No	2012
NCT02415413	Carfilzomib, lenalidomide, dexamethasone followed by autologous transplant	Yes	2019
Musto et al,[34] 2000	Pamidronate	Unclear	2000

Appendix VI. Studies Detailing CRAB Progression.

Trial ID (Author)	CRAB Results Reported
D'arena et al,[30] 2011	Pamidronate group: 39.2% SRE, 37.4% Anemia, 10.7% renal failure, 7.1% extramedullary disease, M-component increase 10.7%
Musto et al,[31] 2008	At the time of progression, SREs were significantly lower in the zoledronic acid group (n = 20 patients; 55.5%) than in the control group (n = 29 patients; 78.3%; odds ratio, 2.90; 95% CI, 1.04-8.06; P = .041) whereas anemia, renal failure, and extramedullary disease were not statistically different between the 2 groups
Lonial et al,[14] 2020	Lenalidomide group: 0% Hypercalcemia, 57.1% Anemia, 0% Renal failure, 42.9% Bone lesion/soft tissue plasmacytoma
Kazandjian et al,[43] 2021	The median clinical PFS objective (development of multiple myeloma) was not reached, as only 2 patients developed multiple myeloma (both developed osteolytic lesions off treatment)
Landgren et al,[28] 2020	Most progression events were SLiM-based and consisted primarily of serum FLC ratio ≥100 or more than one focal lesion by MRI. Two progression events, lytic lesions, were CRAB-based
Martin et al,[66] 2002	After 12 cycles, the M-component had increased in three patients, remained stable in eight and had decreased in one. Hemoglobin levels decreased in the three patients with disease progression. Bone alkaline phosphatase and serum osteocalcin significantly decreased in most patients after four cycles and then remained stable
Musto et al,[34] 2000	After a median follow-up of 36 months (range 12-60), there have been 9 progressions to overt MM in the PMD group. The same number of progressions (9) was seen within the control group. However, among 18 patients who required chemo-radiotherapy for progressive disease, osteolytic bone lesions developed in 7/9 controls, but only in 3 out of 9 of PMD treated patients

Abbreviations: CRAB, hypercalcemia, renal failure, anemia, bone lesions; FLC, free light chain; PFS, progression-free survival; PMD, pamidronate; SLiM, ≥60% clonal plasma cells in bone marrow, involved/uninvolved light chain ratio ≥100, MRI with >1 focal marrow lesion; SRE, skeletal-related events
